# Measles antibody levels among vaccinated and unvaccinated children 6–59 months of age in the Democratic Republic of the Congo, 2013–2014

**DOI:** 10.1016/j.vaccine.2019.09.047

**Published:** 2020-02-24

**Authors:** Hayley R. Ashbaugh, James D. Cherry, Nicole A. Hoff, Reena H. Doshi, Vivian H. Alfonso, Adva Gadoth, Patrick Mukadi, Stephen G. Higgins, Roger Budd, Christina Randall, Guillaume Ngoie Mwamba, Emile Okitolonda-Wemakoy, Jean Jacques Muyembe-Tamfum, Sue K. Gerber, Anne W. Rimoin

**Affiliations:** a Department of Epidemiology, Fielding School of Public Health, University of California, Los Angeles, Los Angeles, CA 90095, United States; b David Geffen School of Medicine at UCLA, Los Angeles, CA 90095, United States; c McKing Consulting, Atlanta, GA 30341, United States; d Kinshasa University, School of Medicine, Kinshasa, The Democratic Republic of the Congo; e Lentigen Technology, Incorporated, Gaithersburg, MD 20878, United States; f DYNEX Technologies Incorporated, Chantilly, VA 20151, United States; g Expanded Program on Immunization, Ministry of Public Health, Kinshasa, The Democratic Republic of the Congo; h Kinshasa School of Public Health, Kinshasa, The Democratic Republic of the Congo; i National Institute for Biomedical Research, Kinshasa, The Democratic Republic of the Congo; j Bill and Melinda Gates Foundation, Seattle, WA 98109, United States

## Abstract

**Background:**

Measles is endemic in the Democratic Republic of the Congo (DRC), and 89–94% herd immunity is required to halt its transmission. Much of the World Health Organization African Region, including the DRC, has vaccination coverage below the 95% level required to eliminate measles, heightening concern of inadequate measles immunity.

**Methods:**

We assessed 6706 children aged 6–59 months whose mothers were selected for interview in the 2013–2014 DRC Demographic and Health Survey. History of measles was obtained by maternal report, and classification of children who had measles was completed using maternal recall and measles immunoglobulin G serostatus obtained from a multiplex chemiluminescent automated immunoassay dried blood spot analysis. A logistic regression model was used to identify associations of covariates with measles and seroprotection, and vaccine effectiveness (VE) was calculated.

**Results:**

Out of our sample, 64% of children were seroprotected. Measles vaccination was associated with protection against measles (OR: 0.15, 95% CI: 0.03, 0.81) when administered to children 12 months of age or older. Vaccination was predictive of seroprotection at all ages. VE was highest (88%) among children 12–24 months of age.

**Conclusion:**

Our results demonstrated lower than expected seroprotection against measles among vaccinated children. Understanding the factors that affect host immunity to measles will aid in developing more efficient and effective immunization programs in DRC.

## 1 Introduction

Measles is a highly contagious, primarily childhood, viral disease caused by a single stranded RNA paramyxovirus (genus *Morbillivirus*) [1], and humans are the natural hosts of measles virus. Eighty-nine to ninety-four per cent herd immunity is required to halt measles transmission [2], and in 2017, there were 173,330 reported measles cases and 109,638 estimated deaths, with a worldwide estimated measles vaccine (MV) coverage of 85% [3]. This is lower than the ≥95% coverage required to eliminate measles [4], and much of the World Health Organization (WHO) African Region, including the Democratic Republic of the Congo (DRC), has even lower coverage than this worldwide average [5].

Measles is endemic in the DRC [5]. Currently, DRC gives one routine dose of measles vaccine to children nine months of age, and in outbreak settings, to children as young as six months. Although the WHO states that all countries should include a second routine dose of MV, regardless of national routine coverage level of the first dose [4], this recommendation has not been implemented in the DRC. Because coverage achieved through healthcare is low in the DRC, attempts are made to reach missed children through Supplementary Immunization Activities (SIA), which Doshi et al. found to be associated with decreased measles incidence [6]. The ability of an infant to seroconvert is age-dependent due to level and decay of maternal antibodies and immunological development; regional differences in seroprevalence have been observed.

Expectant mothers in endemic areas may be more likely to have had natural measles infection, resulting in higher measles antibody levels, and so pass on higher levels of measles antibody transplacentally to their infants, resulting in longer lasting protection than would occur in expectant mothers with vaccine-induced antibody [7], [8]. Children in measles-endemic regions are also at risk of exposure to measles at an earlier age, and this must be considered when determining ideal age of vaccination [4]. Determining drivers of seropositivity and low vaccine effectiveness (VE) is complex and can depend on immunization program logistical capacity [9], vaccine potency [10] and host factors, particularly immune system robustness as a function of development (age) and nutritional status [11], [12].

Measles has occurred even in well-vaccinated populations, raising questions of why adequate protection is not achieved in such groups [13], [14], [15], [16], [17]. While vaccination induces humoral and cellular immune responses similar to those caused by natural disease, the resulting antibody levels are lower among those with vaccine - induced versus natural immunity. As measles continues to be inadequately controlled in DRC [18], [19], population assessment of measles immunity is needed. This study reports measles immunoglobulin G (IgG) antibody levels among children 6–59 months of age participating in the 2013–2014 Demographic and Health Survey (DHS) in the DRC, with the goal of describing population seroprotection, VE, and factors associated with decreased protection against measles.

## 2 Methods

### 2.1 Data source and study population

The 2013–2014 DHS, a nationally representative survey based on a stratified two-stage cluster design, occurred from November 2013 to February 2014. The first stage of the survey consisted of Enumeration Area (EA) formation in which a stratified sample of geographic locations, or clusters (n = 540), was selected with proportional probability according to size. The second stage involved sampling households from each EA; complete listings of households were created within each cluster, and households (n = 9000) were selected with equal probability [20], [21], [22], [23]. Within the selected households, individuals interviewed included 18,827 women aged 15–49 years (all selected households) and 8656 men aged 15–59 years (50% of selected households). The DHS collected biomarker data only on children from households in which men were interviewed. In our dataset, there were 7016 children aged 6–59 months with anthropometric and measles IgG serology data available, and of these, 6706 children had data on measles infection and all other covariates of interest available (approximately 4.5% of children were missing data on covariates of interest and so were excluded from analyses).

Information was collected on weight, health outcomes, vaccination history, and vaccine-preventable disease serology. After parental consent, dried blood spots (DBS) were collected from participants to assess immunity to vaccine-preventable diseases and processed at the University of California, Los Angeles (UCLA) – DRC laboratory at the National Laboratory for Biomedical Research (INRB) in Kinshasa. All survey data were transferred from paper questionnaires to an electronic format using the Census and Survey Processing System (U.S. Census Bureau, ICF Macro). Data were double entered and verified by comparison of both datasets.

Vaccination was reported in three ways. At the time of interview, if mothers possessed a health care worker (HCW)-provided vaccination card indicating the date the child was vaccinated for measles, this was considered a “dated card” report. These reports were only collected from children at least nine months of age, although children 9–59 months of age with a dated card report indicating vaccination at less than nine months of age were included in the “age at vaccination” analyses. If mothers possessed a vaccination card that was marked to indicate measles vaccine was administered, but lacked the date of vaccine administration, this was considered a “marked card” report. Finally, children with caregivers not presenting a card at the time of interview, yet reporting that the child had been vaccinated for measles, were grouped into the “maternal recall” report category. There were no dates associated with this type of vaccination report. Children classified as “unvaccinated” in this study were those reported as such in the 2013–2014 DHS. Additionally, among those with vaccination cards, children who had had measles (as defined by study inclusion criteria) but later received measles vaccination were then re-classified as “unvaccinated” since vaccination occurred after infection (7 children were so classified).

### 2.2 Laboratory analysis

DBS samples were extracted using a modified extraction protocol [24] and, as mentioned previously, processed at the UCLA-DRC laboratory at the INRB. The DBS extraction and assay protocol and multiplex technology have been described elsewhere [25], [26]. Briefly, a 0.25″ DBS punch was extracted, shaking at room temperature, in 1 ml phosphate buffered saline, 0.05% Tween-20, and 5% dried milk, which represents a 1:143 fold dilution assuming 7 µl of serum per punch. The DYNEX Multiplier chemiluminescent automated immunoassay platform with a research use only SmartPLEX assay for measles, mumps, rubella, varicella-zoster virus, and tetanus (MMRVT) (DYNEX Technologies, Chantilly, Virginia) was used to test samples for IgG antibody response (seroprotective level of measles antibody is generally considered to be at least 120 mIU/mL [27]). Polystyrene beads coated separately with antigen to measles, mumps, rubella, varicella-zoster, and tetanus were immobilized within 54-well SmartPLEX assay strips with 10 beads per well and processed using a prototype DYNEX Multiplier® chemiluminescent automated immunoassay instrument. Assay Score (AS) was calculated as a ratio to a five-donor, pooled positive control (PPC) included in each run. The DYNEX chemiluminescent multiplexed immunoassay should be correlated with the plaque reduction neutralization test (PRNT) in order to definitely correlate International Units with AS, but because this was outside the scope of this project, we have reported antibody according to AS. The Limit of Quantitation (LOQ), representing the detection of specific assay response as AS versus PPC, was calculated to be 0.03 AS, and this value, in supplement to maternal report of measles, was used as an additional inclusion criterion for classifying previous measles infection among children with maternally reported history of measles. We utilized the LOQ in conjunction with maternal report in order to decrease misclassification of maternal reports of measles disease, yet avoid missing cases of infants who may have had measles but did not display a robust immune response. This approach, including analyses for internal validation, has been described in previous work [28]. All measles reports in this study are classified as such by utilizing both maternal report and serologic criteria. The cutoff for seroprotection was determined to be 0.14 AS, as determined by validation of the multiplex MMRV serology panel against US-Food and Drug Administration-cleared commercial kits [26]. For purposes of this manuscript, we use the term “seroprotection” to refer to the laboratory cutoff that we are applying to the group under study, while we consider “immunity” to reflect the individual host’s competence to resist measles infection.

### 2.3 Covariate selection

A priori confounders such as age and sex were included in descriptive and regression analyses, as well as other potential confounding variables identified in the literature. Because we previously found associations of vaccination status among the most disadvantaged children compared to the rest of the population [29], we dichotomized variables such as stunting status and wealth index in this way in order to detect potential associations with seroprotection. Because seven or more years of maternal education was found by Hobcraft et al. to be protective against child death [30], this was also included. Low parity was found by Hobcraft to be protective of child death, but in order to better capture the potential exposures from siblings to an individual child, we compared firstborn children to all others as a binary covariate. Although breastfeeding has shown protective associations against child death, because the DHS breastfeeding variable identifies children currently breastfeeding, breastfed in the past, or never breastfed, and the first two categories are strongly tied to age, we did not include this variable in regression analyses. Finally, we included an interaction variable of wealth index x residence in regression analyses to account for the DHS wealth index variable utilizing items more frequently found in urban than rural residences to classify wealth [31]. Collinearity diagnostic statistics were performed on all explanatory variables included in the final regression models, with acceptable results (tolerance for all variables was ≥0.75, variance inflation factor for all variables was ≤1.32) for all variables of interest.

### 2.4 Statistical analyses

Descriptive statistics were performed, and AS geometric mean concentration (GMC) was examined by vaccination report and measles disease history. To assess protectiveness of vaccination and covariates associated with infection, we examined measles cases by age vaccinated (utilizing vaccination report via dated card versus the unvaccinated, n = 2364), year of vaccination (limited to vaccination reports via dated card, n = 738), and nutrition status (stunting) (utilizing the full dataset, n = 6706). We also calculated VE, stratified by age, and limited to vaccination reported via dated card versus the unvaccinated for children 9–59 months of age (n = 2364). Multivariate logistic regression was used to examine both measles infection and seroprotection predicted by vaccination status (limited to vaccinations reported via dated card versus the unvaccinated). To control for variability in probability of selection and interview, DHS stratum, cluster, and individual sampling weights were applied to analyses.

All analyses were performed using SAS software, Version 9.4 (SAS Institute, Cary, NC). Ethical approval was obtained at UCLA Fielding School of Public Health, the Kinshasa School of Public Health and the U.S. Centers for Disease Control and Prevention. As children were younger than the standard age of assent, the parent or guardian of participating children provided consent on the child’s behalf.

## 3 Results

Out of the sample of 6,706 children 6–59 months, 605 (9%) reported a history of measles and 4264 (64%) were seroprotected against measles. Seroprotection varied by vaccination status and report, with 581/738(79%) of children with vaccination report via dated card, 84/113 (74%) of children with vaccination report via marked card, 2738/3896 (70%) of children with vaccination report via maternal recall, and 861/1959 (44%) of unvaccinated children seroprotected (p < 0.0001, Wald Chi-Square Test). Prevalence of seroprotection varied by child age at the time of interview (Table 1), regardless of whether a child was vaccinated and how vaccination was reported, with older children showing higher proportions seroprotected. While not all children with a history of measles were seroprotected, interestingly, 40% of the unvaccinated with no history of measles were seroprotected. Seroprotection also varied by province, regardless of vaccination status or type of vaccination report. For a number of covariates (residence, wealth index, severe stunting, and maternal education), differences in proportion seroprotected were more pronounced among unvaccinated children or those with dated card vaccination report, while less variation was seen within the groups reporting vaccination via marked card or maternal recall. Breastfeeding status showed differences in seroprotection in all vaccination groups, with currently breastfed children showing a lower proportion seroprotected than children breastfed in the past.

**Table 1:** Seroprotection by basic demographics and vaccination status among children 6–59 months.

Variable	Total	Dated card			Marked card			Maternal recall			Unvaccinated		
		n	SP^1^	%	n	SP	%	n	SP	%	n	SP	%
*Age (months)*													
6–8	359	–	–	–	1	0	0	25	6	24	332	53	16
9–11	412	61	35	57	3	0	0	123	66	54	225	69	31
12–23	1537	229	165	72	42	28	67	772	444	58	494	181	37
24–59	4398	448	381	85	66	57	86	2976	2222	75	908	559	62
p-value^2^		**0.0046**				–			**<0.0001**			**<0.0001**	

*Age at vaccination*													
< 9	98	98	72	73		–	–		–	–		–	–
9–11	537	537	429	80		–	–		–	–		–	–
≥ 12	103	103	80	78		–	–		–	–		–	–
p-value			0.6658										

*Measles*													
Yes	605	37	35	95	13	13	100	391	328	84	163	135	83
No	6101	702	546	78	99	72	73	3505	2410	69	1795	726	40
p-value			**0.0061**			**0.0286**			**<0.0001**			**<0.0001**	

*Sex*													
Male	3348	352	274	78	59	42	71	1965	1343	68	972	419	43
Female	3357	386	307	80	54	43	80	1931	1395	72	986	442	45
p-value			0.6790			0.3291			0.0579			0.536	

*Residence*													
Urban	2067	338	285	84	37	24	65	1210	846	70	499	179	36
Rural	4639	400	296	74	76	60	79	2686	1892	70	1534	682	44
p-value			**0.0230**			0.2498			0.8537			**0.0115**	

*Wealth index*													
Poorest	1482	75	46	61	28	25	89	822	583	71	582	256	44
Poorer	1566	139	104	75	27	19	70	876	581	66	535	260	49
Middle	1398	150	122	81	16	14	88	777	551	71	406	174	43
Wealthier	1258	165	128	78	23	13	57	770	543	71	315	115	37
Wealthiest	1067	209	181	87	19	14	74	650	480	74	195	55	28
p-value			0.0590			0.2044			0.3381			**0.0045**	

*Severe stunting*^3^													
Yes	1616	147	122	83	36	29	81	969	653	67	479	248	52
No	5089	591	459	78	76	55	72	2927	2085	71	1554	614	40
p-value			0.1756			0.4782			0.0921			**0.0028**	

*Maternal education*													
< 7 years	4254	367	284	77	67	49	73	2388	1651	69	1480	671	45
≥ 7 years	2451	371	297	80	46	35	76	1508	1087	72	552	190	34
p-value			0.4959			0.8236			0.1573			**0.0005**	

*Breastfed*^4^													
Never	146	16	9	56	2	2	100	94	65	69	36	17	47
Past (not current)	4283	452	389	86	61	52	85	2845	2105	74	952	558	59
Current	2234	268	181	68	48	29	60	930	546	59	1033	275	27
p-value			**0.0038**			–			**<0.0001**			**<0.0001**	

*Firstborn vs. later born*													
Firstborn	1236	190	156	82	19	16	84	668	498	75	374	180	48
Later born	5470	548	425	78	94	68	72	3228	2240	69	1659	681	41
p-value			0.3727			0.2063			0.0572			0.0684	

*Province*													
Kinshasa	472	103	96	93	5	3	69	294	234	79	70	25	35
Kwango	328	28	21	72	5	3	49	215	124	58	79	27	34
Kwilu	510	19	15	78	9	5	50	359	236	66	123	52	42
Mai-Ndombe	307	19	10	52	–	–	–	208	157	76	79	42	53
Kongo Central	295	39	26	66	10	5	53	183	126	69	63	14	21
Equateur	209	54	45	84	2	2	100	93	78	84	60	32	52
Mongala	225	0	0	0	0	0	0	138	123	89	86	53	62
Nord-Ubangi	105	3	3	100	–	–	–	80	67	84	22	17	76
Sud-Ubangi	322	12	9	77	9	9	100	175	129	74	127	72	57
Tshuapa	136	2	2	89	–	–	–	78	51	65	56	26	47
Kasai	222	20	7	37	1	–	–	121	75	62	80	32	40
Kasai-Central	301	57	33	58	6	6	100	131	82	63	107	40	37
Kasai-Oriental	272	25	15	59	13	7	53	131	72	55	102	37	36
Lomami	332	8	7	83	2	2	100	203	132	65	120	38	32
Sankuru	122	1	0	16	1	0	50	50	29	58	71	26	36
Haut-Katanga	289	25	24	94	4	4	90	191	114	60	68	21	30
Haut-Lomami	154	7	5	63	2	–	–	76	38	51	69	27	39
Lualaba	119	11	7	69	5	4	74	46	21	45	56	18	32
Tanganyka	136	3	3	93	–	–	–	47	24	51	86	39	45
Maniema	224	10	5	51	–	–	–	132	79	59	82	34	41
Nord-Kivu	545	177	153	86	11	10	94	274	232	85	83	46	56
Bas-Uele	147	6	3	50	–	–	–	102	80	79	40	23	57
Haut-Uele	98	6	4	55	2	2	73	64	44	69	26	18	68
Ituri	180	9	8	95	15	14	91	115	95	83	41	29	71
Tshopo	155	5	5	100	3	3	100	81	70	86	66	35	54
Sud-Kivu	502	89	77	86	8	7	1	309	224	73	95	41	43
p-value			–			–			**<0.0001**			**0.0011**	

**Total observations**	**6706**	**738**			**113**			**3896**			**1959**		

1 SP = number of seroprotected individuals.

2 Wald chi-square test. Not performed for subgroups with empty cells.

3 According to National Center for Health Statistics/Centers for Disease Control and Prevention/World Health Organization international references standard for height/age standard deviation.

4 The “breastfed” variable had only 6663 observations.

The AS GMC for the entire sample of children aged 6–59 months was 0.159 (SE: 0.006), and stratified GMC varied across age categories within each type of vaccination report, between vaccination report types, and by measles disease report (Supplementary Table 1). Overall, children with vaccination report via dated card displayed the highest GMC (Supplementary Table 1A), and all vaccination categories displayed the highest GMC within the oldest age groups. All groups under 12 months of age demonstrated GMC values below the seroprotective range in all applicable vaccination categories. Stratifying by reported measles status (Supplementary Table 1B), all vaccination categories showed a higher GMC among children with a history of measles than among those with no history of measles.

Measles reports were examined by age at vaccination (children with dated card vaccination reports versus the unvaccinated), year of vaccination (limited to children with dated card vaccination report), and severe stunting (full dataset) (Table 2). Among children vaccinated at less than 9 months of age and 9–11 months of age, 6% and 5%, respectively, had a history of measles, and among those vaccinated at one year of age or older, 2% had a history of measles. Examination of year of vaccination revealed that a higher percentage of children vaccinated in earlier years had a history of measles. Eleven per cent of severely stunted children had a history of measles versus 8% of children who were not severely stunted with a history of measles (p = 0.0259).

**Table 2:** Measles cases by age vaccinated, year of vaccination, and severe stunting.

Age at time of vaccination (months)^1^	Measles −	%	Measles +	%	Total
Unvaccinated	1464	90	162	10	1626
< 9	93	95	6	6	98
9–11	507	94	29	5	537
≥ 12	101	98	2	2	103

**Total**	**2166**	92	**198**	8	2364
**p-value**^2^			**0.0051**		

*Year of vaccination*^3^					
2009	17	71	7	29	24
2010	102	91	10	9	112
2011	123	93	8	6	132
2012	177	96	7	4	184
2013	273	99	4	1	276
2014	10	100	0	0	10

**Total**	**702**	95	**37**	5	738

*Severe stunting*^4^					
Yes	1438	89	178	11	1616
No	4663	92	426	8	5089

**Total**	**6101**	91	**605**	9	6706
**p-value**			**0.0259**		

1 Limited to children with vaccination report via dated card versus the unvaccinated.

2 Wald chi-square test for independence of row and column variables.

3 Limited only to children with vaccination report via dated card.

4 Utilizing the full dataset (all vaccination report types and the unvaccinated).

Vaccination only showed a protective association against measles in the adjusted logistic regression model when vaccination was given at 12 months of age or older (Table 3) (Crude OR: 0.18, 95% CI: 0.03–0.94; Adjusted OR: 0.15, 95% CI: 0.03–0.81), although vaccination was predictive of seroprotection at all ages of vaccination. Firstborn children were also more likely than later born children to be seroprotected (Adjusted OR: 1.51, 95% CI: 1.13–2.03). Limiting vaccination reports to those with dated card (Table 4), VE was low (59%) among children 9–11 months of age, highest (88%) among those 12–24 months, and lowest among the oldest children (43%).

**Table 3:** Measles disease and measles protective immunity predicted by vaccination (comparing children with dated card vaccination report versus the unvaccinated, n = 2364). Limited to children 9–59 months of age.

Variable	Crude odds ratio and 95% CI	Adjusted odds ratio^1^ and 95% CI
*Measles infection outcome*		
**Measles vaccination (ref = unvaccinated)**		
Vaccination at <9 months of age	0.54 (0.13–2.34)	0.80 (0.19–3.30)
Vaccination at 9–11 months of age	**0.52 (0.28–0.96)**	0.79 (0.37–1.71)
Vaccination at ≥12 months of age	**0.18 (0.03–0.94)**	**0.15 (0.03–0.82)**

**Maternal education**		
Primary versus no education (ref)	**0.55 (0.34**–**0.88)**	0.63 (0.36–1.10)

**Birth order**		
Firstborn versus later born (ref)	1.19 (0.79–1.80)	1.39 (0.89–2.16)

**Malnutrition**		
Severe stunting versus all others (ref)	**1.50(1.02–2.20)**	1.23 (0.85–1.78)

*Seroprotection outcome*		
**Measles vaccination**		
Vaccination at <9 months of age	**2.81 (1.28–6.17)**	**2.46 (1.13–5.35)**
Vaccination at 9–11 months of age	**4.03 (2.85–5.68)**	**3.56 (2.40–5.27)**
Vaccination at ≥12 months of age	**3.42 (1.74–6.73)**	**2.44 (1.10–5.42)**

**Maternal education**		
Primary versus no education (ref)	1.22 (0.94–1.58)	0.91 (0.68–1.23)

**Birth order**		
Firstborn versus later born (ref)	**1.52 (1.16–1.98)**	**1.51 (1.13–2.03)**

**Severe stunting**		
Severe stunting versus all others (ref)	1.26 (0.95–1.66)	1.26 (0.87–1.82)

1 Controlling for the following additional covariates: age, mother's education, sex, rural versus urban residence, wealth index, residence * wealth index interaction, birth order, province of residence, and stunting (according to National Center for Health Statistics/Centers for Disease Control and Prevention/World Health Organization international references standard for height/age standard deviation).

**Table 4:** Vaccine effectiveness stratified by age, limited to vaccination reports via dated card versus the unvaccinated (n = 2364).

Age (months)	No history of measles	History of measles	Total (n)	Attack rate (per 100)^1^, ^2^	Vaccine effectiveness (%)^3^
9–11					
Unvaccinated	216	9	225	4.0	59.0
Vaccinated	60	1	61	1.6	

12–23					
Unvaccinated	458	35	494	7.1	87.7
Vaccinated	227	2	229	0.9	

24–59					
Unvaccinated	791	117	908	12.9	42.8
Vaccinated	415	33	448	7.4	

Total					
Unvaccinated	1464	162	1626	10.0	49.7
Vaccinated	702	37	738	5.0	

1 Attack rate in the unvaccinated calculated as: (cases among the unvaccinated/total unvaccinated) × 100.

2 Attack rate in the vaccinated calculated as: (cases among the vaccinated/total vaccinated) × 100.

3 Vaccine effectiveness calculated as: ((AR_unvaccinated_ − AR_vaccinated_)/AR_unvaccinated_) × 100.

## 4 Discussion

Immunity derived from a single dose of measles vaccine administered at 9 months of age is too low to effectively interrupt measles virus transmission in DRC, with only approximately two-thirds of children demonstrating seroprotection. Previous work suggests that lack of seroprotection could be due to lack of vaccination, failure to mount an effective immune response, loss of vaccine integrity (via breaks in the cold chain or other means), incorrect vaccine administration, vaccination failure, host genetic factors, or infant infection during which maternal antibody interference prevents a robust immune response [14], [32], [33], [34], [35].

Seroconversion GMC did not reach the seroprotective range for the age groups under 12 months, regardless of vaccination status or report type. Children who had been vaccinated at 6–8 months of age and 9–11 months of age demonstrated the highest percentage of measles reports among those reporting vaccination via dated card (6% and 5%, respectively) in our analysis (although most of these reports occurred when the child was nine months of age or older). As stated previously, 6–8 month infants are below the recommended age for the first dose of measles vaccine, yet are at risk of measles infection [36], [37], [38]. In outbreak settings, vaccination is made available to infants 6 months of age and older, however this is a short eligibility period and unlikely to have affected many of the children in the study. Finally, as measles outbreaks are episodic and seasonal, the nationwide outbreaks of 2011–2012 would have infected the 12–24 month cohorts in the 2013–2014 DHS, whereas the infants who were born after the outbreak had a lower risk of measles exposure, which may contribute to the difference in immunity observed between these ages [39]. Further research is warranted to determine the impact of seropositivity due to natural infection or vaccination among infants and to inform recommendations on the timing of the first dose of measles in endemic measles settings such as DRC.

Among children vaccinated from 9–11 months of age, vaccination did not have a protective association against measles in the adjusted logistic regression model. Further, VE at 59% was suboptimal and even below the WHO African Region report of a median VE of 73% [40]. Measles vaccination at 9 months of age is thought to have a 10–15% vaccination failure rate [41], and one U.S. study found a measles attack rate of 6.3% in children vaccinated prior to 12 months of age (attack rate in unvaccinated children was 8.5%, and 1.7% in children vaccinated at ≥12 months of age), with vaccine failure determined to play a meaningful role in this outbreak [16]. Although nine months is the recommended age for the first routine dose of measles vaccine in DRC, a single dose administered at 9 months does not appear to induce adequate protection against measles in our study population.

Children one year of age or older was the only group showing a protective association of vaccination against measles in the adjusted logistic regression model. VE in our analysis was found to be highest among children 12–24 months of age (88%), and this differs somewhat from the Doshi et. al findings, examining measles cases from case-based surveillance in the DRC [9]. Low VE might indicate improper vaccine administration, underreporting of disease, vaccination misclassification, or vaccine failure. Reasons for lower VE in older age groups should be further explored. Additionally, VE may have been affected by children vaccinated via SIAs yet reported as unvaccinated in the DHS, and such misclassification could have driven the VE closer to the null value.

There are a few findings that warrant additional comment. First, 40% of the unvaccinated who did not have measles did have seroprotective measles antibody values (Table 1), suggesting that measles illness could have gone unrecognized (perhaps during the extensive measles outbreaks in 2011–2012), that vaccination due to SIA might have occurred among children classified as unvaccinated by the DHS, or that vaccination cards might have underestimated coverage, as has been found in low-middle and high-income countries [42]. Second, 16% and 17%, respectively, of the maternal recall and unvaccinated groups had a history of measles yet did not have protective levels of measles antibody. This suggests that some reported measles was in fact not measles and perhaps rubella or other viral exanthems. Regarding serologic trends according to breastfeeding status, in general, children breastfed in the past demonstrated a higher percentage of individuals seroprotected versus children currently breastfed. We suggest that this is at least partially explained by age, with children breastfed in the past more likely to be older and vaccinated. Neither wealth index nor the wealth index x residence interaction variable were statistically significant predictors in the regression models.

There was a statistically significant association between severe chronic malnutrition (stunting) and measles (p = 0.0259). Stunting is chronic in nature, and was only measured at the time of the DHS study, while measles occurred in children at various timepoints preceding the study. For this reason, although we cannot say whether severe stunting increased risk of measles, it is a strong indicator of previous poor nutritional status which is associated with the depression of cell-mediated immunity and known to impact susceptibility to infectious diseases in children [11], [12]. While severely malnourished children showed higher prevalence of measles compared to all other children, malnutrition was not a significant predictor in the regression analyses limited to vaccination reports via dated card, and this may have been due to any impact of malnutrition on measles infection being explained by other covariates in the model for this group of children.

Strengths of this study include a large, nationally representative sample and increased confidence in maternal measles reports due to confirmation of measles serology via multiplex assay. To our knowledge, this is the first study to report national measles seroprotection status of children in DRC. Classification of vaccination status and measles history were the core challenges of this study. First, potential residual misclassification of vaccination status, particularly among the maternal recall vaccination reports, was a concern. If misclassification of vaccine status occurred, it was likely differential, since children with vaccination cards were more likely to have correctly classified vaccination status than those who reported via maternal recall. Differential misclassification could result in estimates biased either toward or away from the null. To address this concern, we limited a number of analyses to vaccinations reported via dated card. A second limitation in terms of vaccination classification is our lack of individual-level data for measles SIAs. It is possible that a child may have had an additional dose of measles vaccine that was not reported as part of the DHS. Similarly, we were only able to verify a single dose of measles vaccination through DHS vaccination data, but via SIA, some children may have received a second dose. Thirdly, as discussed in previous work [28], laboratory-confirmed diagnosis of measles was not available, and so we could not fully verify that children classified as having measles did not have measles antibody due to vaccination alone. Although clinical signs of measles can support a higher positive predictive value in measles endemic areas (when laboratory confirmation is not available) [43], measles reports were obtained from mothers rather than trained health care workers, and so may be less reliable. However, other work utilizing household surveys in sub-Saharan Africa has shown that maternal report can be a valid measure for events surrounding child illness [43]. In addition to maternal recall, we were able to substantiate reports of measles with child serologic data, therefore lessening the possibility of outcome misclassification due to maternal recall. Finally, if misclassification of measles status occurred, this might have made the VE estimates appear falsely low, as false positive maternal reports among the unvaccinated might have been excluded for not meeting the IgG criterion of 0.03 AS, but false positive maternal reports among the vaccinated might have been categorized as measles cases by meeting the 0.03 AS criterion due to vaccine-induced antibody.

## 5 Conclusion

This study identifies clear gaps in measles seroprotection that can inform future policy and research. Our results demonstrate that a single vaccination at 9 months of age (or earlier) is associated with significant vaccination failure contributing to inadequate seroprotection in DRC. Population immunity against measles can be improved by expanding measles vaccination coverage, ensuring that vaccination results in adequate immunity to protect against measles infection, and incorporating a second dose of measles vaccine into the routine vaccination schedule, as recommended by WHO. Understanding factors that decrease host seroprotection and increase risk of vaccine failure will aid in developing more efficient and effective immunization programs.

## Funding

This work was supported by the Faucett Catalyst Fund and the Estimating Population Immunity to Poliovirus in the Democratic Republic of the Congo Grant by the Bill and Melinda Gates Foundation [Grant No. OPP1066684].

## Disclaimers

The views expressed here are the authors’ alone and do not reflect the official policy or position of the Department of the Army, Department of Defense, or the U.S. Government.

## Declaration of Competing Interest

The authors declare that they have no known competing financial interests or personal relationships that could have appeared to influence the work reported in this paper.
