# Supplementary material for: Measles antibody levels among vaccinated and unvaccinated children 6–59 months of age in the Democratic Republic of the Congo, 2013–2014
Source: Vaccine. 2020 Feb 24;38(9):2258–65. doi: 10.1016/j.vaccine.2019.09.047 (PMC7026690; doi:10.1016/j.vaccine.2019.09.047)
Supplement: Supplementary data 1 [file mmc1.docx]

| Supplementary Table 1A. Measles antibody Geometric Mean Concentration (GMC) by vaccination status and age among children 6-59 months.^1^ | | | | | | | | | | | | | |
| --- | --- | --- | --- | --- | --- | --- | --- | --- | --- | --- | --- | --- | --- |
| **Age (months)** | **n** | **Dated card** | | | **Marked card** | | | **Maternal recall** | | | **Unvaccinated** | | |
|  |  | **n** | **GMC** | **SE** | **n** | **GMC** | **SE** | **n** | **GMC** | **SE** | **n** | **GMC** | **SE** |
| 6-8 | 359 | --- | --- | --- | 1 | **0.017** | **0.001** | 25 | **0.041** | **0.015** | 332 | **0.022** | **0.003** |
| 9-11 | 412 | 61 | **0.09** | **0.034** | 3 | **0.006** | **0.003** | 123 | **0.085** | **0.015** | 225 | **0.036** | **0.007** |
| 12-23 | 1537 | 229 | 0.215 | 0.025 | 42 | **0.137** | **0.055** | 772 | **0.131** | **0.012** | 494 | **0.052** | **0.007** |
| 24-35 | 1556 | 211 | 0.256 | 0.043 | 31 | 0.227 | 0.094 | 983 | 0.19 | 0.014 | 331 | **0.121** | **0.017** |
| 36-47 | 1433 | 117 | 0.409 | 0.048 | 20 | 0.384 | 0.097 | 979 | 0.268 | 0.019 | 317 | 0.179 | 0.018 |
| 48-59 | 1408 | 120 | 0.361 | 0.047 | 15 | 0.396 | 0.100 | 1014 | 0.315 | 0.019 | 260 | 0.239 | 0.029 |
| **Total** | 6706 | 738 | 0.253 | 0.021 | 113 | 0.195 | 0.043 | 3896 | 0.212 | 0.01 | 1959 | **0.074** | **0.005** |
| ^1^Bolded values indicate GMC below the seroprotective range. | | | | | | | | | | | | | |

| Supplementary Table 1B. Measles antibody Geometric Mean Concentration (GMC) by vaccination and reported measles status.^1^ | | | | |
| --- | --- | --- | --- | --- |
|  | **Measles -** | | **Measles +** | |
| **Vaccination status** | **n** | **GMC (SE)** | **n** | **GMC (SE)** |
| Dated card | 702 | 0.246 (0.020) | 37 | 0.429 (0.095) |
| Marked card | 99 | 0.169 (0.040) | 13 | 0.559 (0.218) |
| Maternal recall | 3505 | 0.194 (0.009) | 391 | 0.472 (0.051) |
| Unvaccinated | 1795 | **0.062 (0.004)** | 163 | 0.534 (0.102) |
| ^1^Bolded values indicate GMC below the seroprotective range. | | | | |
